# Characterization of a Cell-Culturing System for the Study of Contact-Independent Extracellular Vesicle Communication

**DOI:** 10.5772/62580

**Published:** 2016-02-26

**Authors:** Anne Louise Schacht Revenfeld, Evo Kristina Lindersson Søndergaard, Allan Stensballe, Rikke Bæk, Malene Møller Jørgensen, Kim Varming

**Affiliations:** 1 Department of Clinical Immunology, Aalborg University Hospital, Aalborg, Denmark; 2 Department of Health Science and Technology, Laboratory for Medical Mass Spectrometry Fredrik Bajersvej, Aalborg University, Aalborg, Denmark

**Keywords:** Extracellular Vesicles, Cell Communication, Contact-independent, EV Array, Phenotype, Transwell

## Abstract

Appropriate and well-documented *in vitro* cell-culturing systems are necessary to study the activity and biological function of extracellular vesicles (EVs). The aim of this study was to describe an experimental system, in which dynamic, vesicle-based cell communication can be investigated. A commercially available cell-culturing system was applied to study contact-independent cell communication, which separated two cell populations using a membrane with a pore size of 0.4 μm. The EV exchange characteristics between the two compartments in the culture set-up was preliminarily investigated in a cell-free set-up, and analysed using the Extracellular Vesicle (EV) Array and Nanoparticle Tracking Analysis. The application of the cell-culturing set-up was demonstrated using co-cultures of human primary cells. The effects of the relative placement of the two cell populations on the phenotype of EVs found in the cell supernatant were investigated. The results indicate that this placement can be important for the biological hypothesis that is being investigated. These observations are relevant for short (<24h) as well as long (several days) studies of vesicle-based cell communication. Moreover, the introduced cell-culturing set-up and analytical strategy can be used to study contact-independent vesicle communication in a reproducible manner.

## 1. Introduction

It is currently well accepted that extracellular vesicles (EVs) are released from a plethora of cell types in many biological systems [1]. Furthermore, these vesicular entities can be used as mediators of intercellular communication by a cargo of proteins and RNAs [2]. In line with this, EVs play an important role in many cellular processes in humans, both in physiological and pathophysiological scenarios [1–3]. However, uncovering the specific biological functions of EVs necessitates well-documented approaches before the carrying out of valid functional studies. Many *in vitro* experiments carried out to answer these biological questions use two different approaches: i) EVs from one cell population/condition are isolated and added to another cell population [4–7]. Subsequently, the effect of the EVs on the second cell population is investigated. ii) The two cell populations are co-cultured, but are separated by a membrane with a pore size that allows for the transport of vesicles of a defined size range, as well as soluble factors [4, 8, 9]. The effects of the EV- and signal molecule-based communication can thereafter be determined for either one or two cell populations. The latter set-up represents the most dynamic of the two approaches for studying vesicle-based communication, since it is based on co-cultures. Consequently, it incorporates the continuous communication between the studied cells, with a consequent greater resemblance to the *in vivo* conditions.

**Figure 1.**
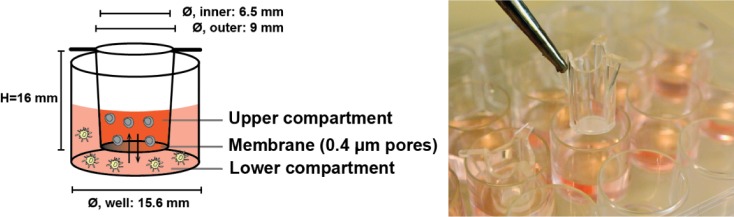
**Principle of the cell-culturing system for contact-independent cellular communication**. The use of cell culture inserts was applied in order to create a two-compartment cell-culturing system. The insert was placed within a well of a standard 24-well culture plate, creating an upper compartment (UC) and a lower compartment (LC). Multiple flanges at the top of the insert suspended it onto the edge of the culture plate well, ensuring the lack of direct contact between the insert and the well. An incorporated membrane in the bottom of the insert facilitated the separation of the compartments. The membrane pore size of the applied inserts was 0.4 μm in diameter, thus allowing for the passage of smaller vesicle subsets, as well as soluble factors (indicated by the arrows). The pore density was 1 × 10^8^ pores/cm^2^, while the effective membrane area was 33 mm^2^.

The cell-culturing set-up described in the current study is based on the co-culture approach introduced above. The principle of this set-up is depicted in Figure 1. Several factors need to be taken into account when designing such a study. A key factor, which this study investigates, is the importance of which cell populations are placed in the upper compartment (UC) and the lower compartment (LC), relative to the biological hypothesis being tested. This is relevant, since we were able to demonstrate that this placement can affect the experimental outcomes, both for short (<24h) and long studies (several days) of vesicle-based cell communication. Additionally, the subsequent analysis of the EVs must be reliable, reproducible and provide as much information as possible. The aim of this report is to present a combined cell culturing and analytical set-up, which allows for an easy and reproducible detection of differences in EV phenotypes caused by contact-independent cellular communication.

## 2. Materials and Methods

### 2.1 Transmembrane exchange of EVs

*Cell culture conditions and EV collection*: Cell-free and EV-enriched conditioned cell media were obtained from the human colon-cancer cell line LS180 (ATCC® CL-187™; ATCC, Manassas, VA, USA) and cultured in a growth medium containing RPMI 1640 (Gibco, Life Technologies, Carlsbad, CA, USA), 10 % ultracentrifuged (100.000 xg, 24 h, 4 °C; Ti45 rotor, Beckman Coulter, Brea, USA) heat-inactivated foetal bovine serum (FBS) (Gibco), 100 U/mL penicillin and 10 μg/mL streptomycin (Ampliqon, Odense, DK)). To remove the cells, the conditioned media were centrifuged at 500 xg, 5 min, RT, after collection. To the cell-free supernatant, a protease inhibitor cocktail was added (EDTA-free, Roche, Basel, Switzerland, diluted 1:50 in PBS). Subsequently, the EV-rich supernatant was reduced in volume by using a 15 mL Amicon® Ultra filter unit with a 100k MWCO (Merck Millipore, Darmstadt, Germany). The volume-reduced, EV-rich supernatant was washed twice with PBS prior to use. The final volume of the cell supernatant was approximately 1/20 of the original volume.

*Dilution series to investigate transmembrane exchange of EVs*: The following dilutions of the EV-rich supernatant from LS180 were included: undiluted, 1:10, 1:50, 1:100, 1:500 and 1:1000. All dilutions were made with the growth medium. Initially, 800 μL of the supernatant was placed in a well in a 24-well plate (Nunc, Thermo Scientific, Waltham, MA, USA). This compartment was designated as the LC (Figure 1). Subsequently, 400 μL of growth medium was put in the UC, constituted by a Millicell® Hanging Cell Culture Insert (#PIHT 12R 48, Merck Millipore). With this set-up, the exchange of EVs from the LC to the UC was investigated. To describe the transmembrane EV exchange from the UC to the LC, the reverse set-up was made (i.e., growth medium in the LC; EV-rich supernatant in the UC). The culture plate holding the inserts was placed in a CO_2_-incubator (temperature: 37 °C; CO_2_-concentration: 5 %; relative humidity: 90 %) for 24 hours with no agitation. After this, the contents of each compartment was harvested into separate tubes and stored at −40 °C until semi-quantification of the vesicles by the EV Array. No further isolation of the EVs was performed.

### 2.2 Contact-independent cell communication

*Isolation of cells*: Buffy coats were obtained from healthy blood donors at the Aalborg University Hospital blood bank. Each blood donor had signed a written consent form, allowing the use of his or her blood for research purposes. The procedure was approved by the local ethics legislation. Isolation of peripheral blood mononuclear cells (PBMCs) was accomplished by using gradient centrifugation with a Lymphoprep™ (Axis-Shield, Oslo, NO). A final washing step (350 xg, 7 min, RT) was performed to reduce the number of platelets in the final cell suspension. The PBMCs were either used directly after the isolation or stored at −140 °C in a storage medium (RPMI 1640, 40 % heat-inactivated FBS, 10 % dimethyl sulphoxide (Merck Millipore), 100 U/mL penicillin and 10 μg/mL streptomycin).

*Cell culture set-up and EV collection*: A co-culture was created with PBMCs from two donors with known HLA serotypes. The PBMCs from one donor were designated the stimulator cells, while the cells from the second donor were entitled the responder cells. Prior to the co-culture, the stimulator cells were irradiated (1700 rad) to inhibit their proliferation. A Millicell® Hanging Cell Culture Insert separated the stimulator cells and responder cells (Figure 1). The UC of the cell-culturing system contained 2.5×10^5^ cells in a total volume of 400 μL, while the lower chamber contained 5×10^5^ cells in 800 μL. Control samples with monocultures of both stimulator and responder cells were also included. Here, 5×10^4^ of either responder or stimulator cells were seeded in a 96-well plate in a total of 150 μL culture medium. The cells were placed in a CO_2_-incubator (temperature: 37 °C; CO_2_-concentration: 5 %; relative humidity: 90 %) for six days. On Day 6, the conditioned cell media were harvested from each compartment separately and centrifuged once at 500 xg, 10 min, RT to pellet cells. A protease inhibitor cocktail (EDTA-free, diluted 1:50 in PBS) was added to the cell-free supernatants prior to storage at −40 °C until vesicle phenotyping by the EV Array or size determination with Nanoparticle Tracking Analysis (NTA) was carried out. No further isolation of the EVs was performed.

### 2.3 EV Array analysis

*Production of microarrays*: Microarray printing was performed on a SpotBot® Extreme Protein Edition Microarray Printer (Sunnyvale, ArrayIt, CA, US), as previously described [10].

*Antibodies/proteins for the phenotyping of vesicles*: For the phenotyping, a total of 10 anti-human antibodies and one protein were used. They are listed in the following with the corresponding product number (#) or clone. From R&D Systems (Minneapolis, MN, USA): CD82 (#423524) and TNFRI (#DY225). From Biolegend: CD63 (MEM-259) and HLA-DR (L243). From LifeSpan BioSciences, Inc. (Seattle, WA, USA): CD9 (#LS-C35418) and CD81 (#LS-B7347). From Abcam (Cambridge, MA, USA): Flotilin-1 (#Ab41927). From Haematologic Technologies, Inc. (Essex Juncton, VT, USA): Lactadherin (#BLAC-1200) (protein). From BD Biosciences: CD3 (Hit3a). From Abbiotec (San Diego, CA, USA): CD11a (HI111). From eBioscience (San Diego, CA, USA): ICAM-1 (R6.5). All antibodies/proteins for the phenotyping were printed in triplicate at 200 μg/mL diluted in PBS containing 5 % glycerol.

*Antibodies for the semi-quantification of vesicles*: For the semi-quantification of vesicles, anti-CD9, anti-CD63 and anti-CD81 were printed on the microarray slides, as previously described [11]. In short, 18 repeated spots were printed using a cocktail of the three antibodies, each in a concentration of 100 μg/mL.

*Catching and visualization*: The entire procedure was performed as described previously [11]. In brief, the printed slides were blocked, incubated with the EV-containing sample, followed by the detection of bound EVs with biotinylated anti-CD9, -CD63 and -CD81 and, subsequently, Cy5-labelled streptavidin.

**Figure 2.**
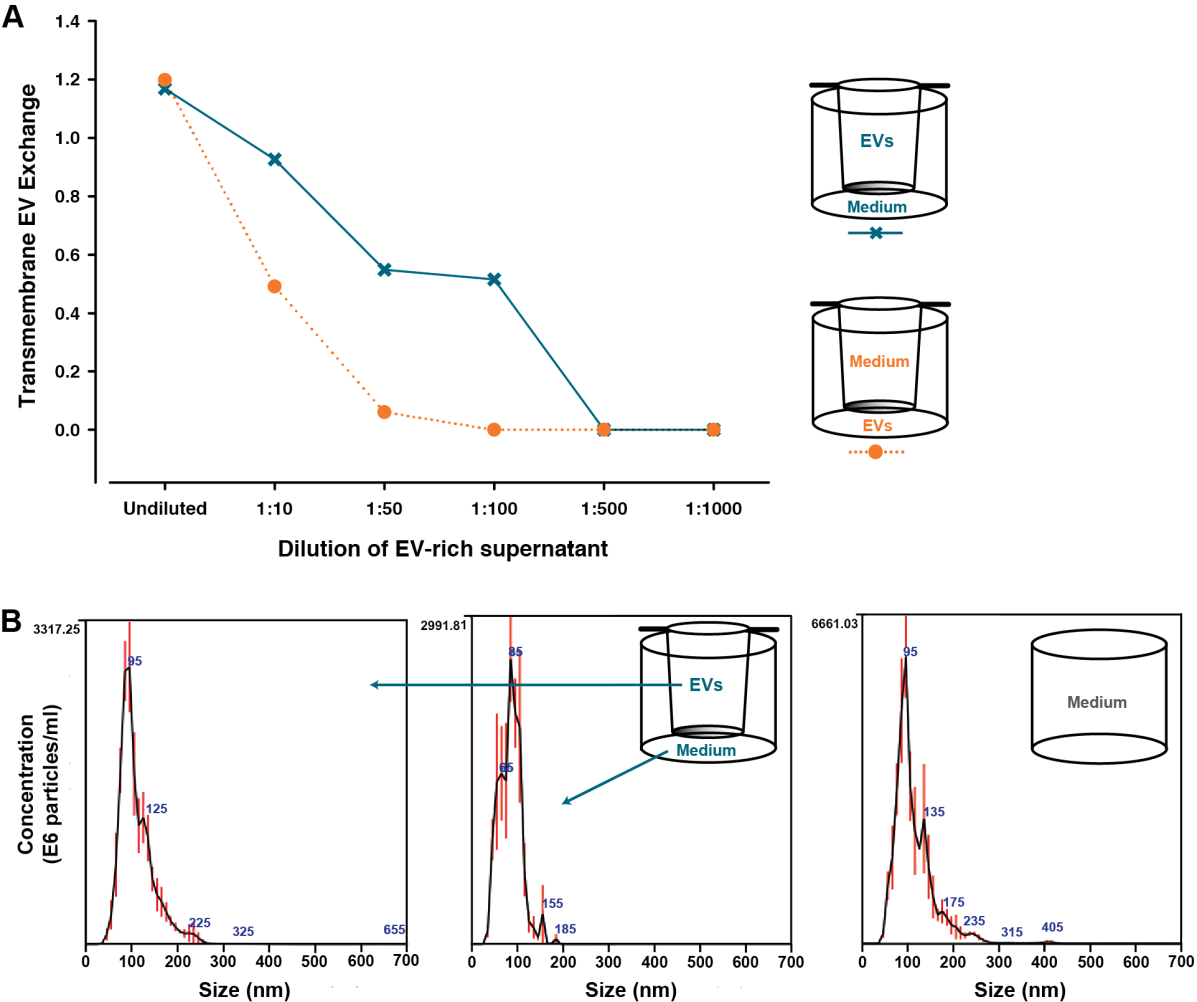
**Transmembrane exchange of EVs between the two compartments of the cell-culturing set-up**. The exchange of EVs from the UC to LC of the cell-culturing set-up, and vice versa, was evaluated. **A**) An EV-rich, cell-free supernatant from the human cell line LS180 was placed in one of the two compartments, while growth medium was placed in the opposite compartment. This was done with six dilutions of the supernatant, as indicated on the x-axis. After 24h, the contents of each compartment were harvested, and the amount of CD9, CD63 and/or CD81 was semi-quantified using the EV Array for the EVs that had bound to the array. The transmembrane EV exchange shows the relationship between the EV Array signals from the compartment initially containing growth medium and the compartment, to which the EV-rich supernatant was originally added. **B**) The size distribution of the EVs in the UC and LC of the 1:50 dilution was determined by NTA. Here, the upper to lower samples are shown, while a summary of the remaining samples can be found in Table 1. In addition, the applied growth medium was also analysed separately. For each sample, the histogram indicates the mean of the triplicates. The numbers in blue indicate the particle size at the peak measurements in the histogram, while the red bars indicate +/- 1 standard error of the mean. The x-axis has been truncated from 1000 nm to 700 nm, since no particles were detected in the largest size range.

*Data analysis*: Data analysis and the creation of graphs were carried out using SigmaPlot (version 11, Systat Software Inc, San Jose, CA, USA) and Excel (version 2013, Microsoft, Redmond, WA, USA). For a given antibody spot, the signal intensity was calculated as the mean signal of the triplicate spots (18 spots for semi-quantification) in relation to the sample signal of the negative spot (PBS) in triplicate. For each spot, the signal intensity was calculated by subtracting the mean of the background (no sample/blank, washing buffer) from the mean of the foreground (spot signal). Before visualization and the calculation of linearity, the antibody signal intensities were converted to log space by log2 transformation. The transmembrane EV exchange, evaluated from the semi-quantitative data (Figure 2), was calculated as: EV Array signal in compartment with growth medium/ EV Array signal in compartment with added EV-rich cell supernatant.

### 2.4 Nanoparticle tracking analysis (NTA)

*Instrument details*: For EV-size determination, NTA was performed with a NanoSight LM10-HS system equipped with a finely tuned 405 nm laser (NanoSight Ltd., Amesbury, UK), supplied with the NTA 3.0 0060 analytical software, which was used for capturing and analysing the data. The camera type was EMCCD and the camera level was set to 11.

*Size determination*: The NanoSight was calibrated with 100 nm polystyrene latex microbeads (Thermo Scientific, Fremont, USA) prior to analysis. Dulbecco's phosphate buffered saline (DPBS) without Ca^2^^+^ and Mg^+^, and filtered using a 0.22 μm filter prior to use (Lonza, Verviers, Belgium), was used to dilute the microbeads (1:1000 dilution) and the EV-rich supernatants (1:40 dilution). The samples were manually injected into the sample chamber and a temperature-measuring device inserted directly into the sample chamber was applied to record the temperature of the sample for each run. Samples were measured with a slide shutter of 600 and with a slider gain of 300 for 60 s. The applied dilutions yielded between 20–100 particles/frame and each sample was measured in triplicate. Subsequently, the integrated software automatically processed the data, yielding values such as the mean, the median, the mode particle size, the value of the highest point of the peak and the corresponding standard deviations. The detection threshold was set to three and the blur setting was 9 × 9.

## 3. Results

### 3.1 Transmembrane EV exchange between the two compartments of the cell-culturing set-up

Initially, the exchange characteristics of EVs between the two compartments of the cell-culturing system (Figure 1) were investigated using a cell-free approach. This was done to evaluate whether the experimental outcome was affected by the relative position of the cells in the two compartments. For this purpose, a volume-reduced, EV-rich supernatant from the human colon-cancer cell line LS180 was placed in one of the compartments and a growth medium was placed in the opposite compartment. Consequently, the transport of EVs from the UC to the LC, and in the reciprocal direction, was studied. After 24h, the extent of transmembrane EV exchange was evaluated in each compartment by using the EV Array to semi-quantify the contents of CD9-, CD63- and/or CD81-containing EVs. The results from this analysis can be seen in Figure 2. Here, the transmembrane EV exchange illustrated in Figure 2A designates the ratio between the EV Array signal from the compartment, which initially only contained growth medium, and the signal from the EV starting compartment. From Figure 2A, it can be deduced that the transmembrane EV exchange was greater from the UC to the LC in the investigated time frame, as compared to the reciprocal direction. This difference was already noticeable with the first dilution. Here, the signal from the UC of the lower to upper set-up (orange) was reduced to roughly 50 % of that from the LC, while the signals from both UC and LC of the reversed set-up were almost similar (blue). Furthermore, with the 1:50 dilution, practically no signal could be detected in the UC for the lower to the upper combination. For the reversed combination, this tendency was observed later, at the 1:500 dilution.

As an additional investigation, the size distributions of the EVs present in each compartment were determined by Nanoparticle Tracking Analysis (NTA) after 24h of transmembrane EV exchange. With this investigation, it was possible to evaluate whether both small and large EVs were present in the EV-rich supernatants. The analysed samples included those from the 1:50 dilution (Figure 2). The histograms of the EV size distribution for the upper to lower combination can be seen in Figure 2B. The remaining results from the NTA are summarized in Table 1, in which they can be compared to the corresponding EV Array signals. The samples presented in Figure 2B contained smaller vesicles, with 90 % being smaller than approximately 100 nm and 150 nm for the LC and UC, respectively. Moreover, no larger vesicles/particles (>400 nm) were present in any of the samples (Figure 2B), including the growth medium analysed prior to the investigation of the transmembrane EV exchange. From the mean and mode values found in Table 1, it is not possible to accurately distinguish the transferred EVs from those inherently present in the growth medium. However, two points can be deduced when comparing the NTA data with the matching EV Array signals. First, it appears that even though vesicles were present in the growth medium, despite using UC FCS, these EVs do not elicit a specific and interfering EV Array signal. This consequently leads to the second point, indicating that the EV Array signals detected in the compartments initially only holding growth medium come from a transmembrane exchange of LS180 EVs. The EV Array signal from that compartment was 8.6 fold higher in the upper to lower scenario than in the opposite direction (Table 1). This accordingly supports the tendency deduced from Figure 2A, in which the transmembrane EV exchange was seemingly greatest from the UC to the LC.

**Table 1.** **Size distribution of the vesicles after 24h of transmembrane EV exchange**. An EV-rich, cell-free supernatant was placed in either the UC or LC of the cell-culturing set-up, while growth medium was placed in the opposite compartment. After 24h, the size of the EVs present in each compartment was determined by nanoparticle tracking analysis (NTA). The data presented are from the 1:50 dilution shown in Figure 2. For each compartment, the signal from the EV array is given along with the results from the NTA. The mean and the mode, and the corresponding standard error of the mean (SEM), are given for triplicate measurements of each sample.


	EV array signal	Mean size (SEM) [nm]	Mode (SEM) [nm]

**EV transport: Upper to lower**			
		
UC	7.8	105.9 (5.5)	88.6 (2.6)
LC	4.3	79.3 (2.7)	87.3 (9.1)

**EV transport: Lower to upper**			
		
LC	7.7	102.6 (0.6)	91.8 (5.8)
UC	0.5	97.1 (3.3)	85.3 (5.1)

Growth medium	0	103.9 (3.1)	102.1 (3.7)


### 3.2 Demonstration of the applicability of the cell-culturing set-up and analysis platform

**Figure 3.**
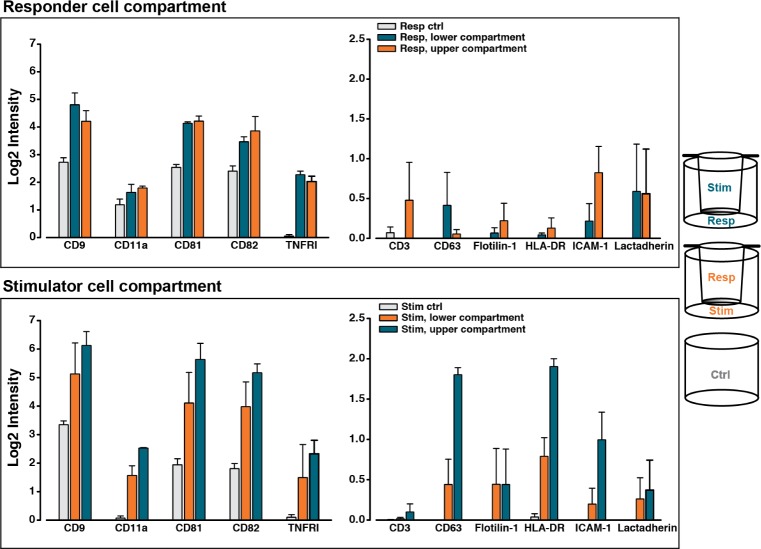
**EV phenotype after the co-culture of human peripheral blood mononuclear cells**. A six-day, contact-independent co-culture between peripheral blood mononuclear cells (PBMCs) from two different individuals was created. The phenotypes of the cell-derived EVs were evaluated using the EV Array to detect differences caused by the relative placement of the two cell populations. Consequently, both combinations of responder (resp) and stimulator (stim) cells in the lower compartment (LC) and upper compartment (UC) were included. For non-specific reactions, monocultures of both stimulator (stim ctrl) and responder cells (resp ctrl) were included. Antibodies targeting the listed markers on the x-axis were used for the capturing of the EVs. The panel of antibodies targeted both immunological markers, which were related to the different leukocyte subsets within the PBMCs, and more general vesicle-related markers. The observed signal for each of the markers implies the simultaneous presence of CD9, CD63 and/or CD81, since a cocktail of antibodies against these three EV markers was used for detection. For each combination of responder and stimulator cells in the UC and the LC, the bars show a mean value ± SEM from two independent experiments using cells from the same two donors. EV Array measurements were performed in triplicate.

In addition to the cell-free set-up, the presented cell-culturing system was further investigated using a co-culture of human peripheral blood mononuclear cells (PBMCs). In this co-culture, one cell population was designated as the stimulator cells, since these cells could induce an immunological response in the responder cells, constituted by the second cell population. The stimulator cells were not subject to a reciprocal activation, since they had been irradiated, making the cell communication oneway. Both combinations of the stimulator and the responder cells in the UC and LC were made and, after six days of co-culturing, the phenotype of the cell-derived EVs was determined, as shown in Figure 3. It can be seen for both combinations of the co-culture that all the reported protein markers were present on EVs above control levels (grey bars). Moreover, the presence of the more EV-specific markers, including CD9, CD81 and CD82, were the most abundant, when compared to the remaining markers included in the EV phenotyping. One exception to this related to CD63, which was only marginally detected in comparison to CD9, CD81 and CD82. The EV Array signals for the majority of the more cell-specific markers were at the lower level of detection for the applied array, with the exception of CD11a and TNFRI. Nonetheless, the relative placement of the two cell populations in the co-culturing set-up affected the obtained EV Array signals for both the vesicle- and cell-specific markers, although to a varying degree. From the responder cell compartments (Figure 3, top panel), some of the most pronounced differences were observed for ICAM-1, with an almost 3.5 fold higher signal for this marker from the EVs harvested from the UC (orange) than for the LC (blue) of the responder cells. Moreover, CD3 could only be detected from EVs in the responder cell compartment in one of the settings. Finally, the detection of CD81 was almost 1.5 times higher in the UC sample, when compared to the LC sample. In contrast, comparable signals for CD81 were observed in both stimulator cell compartments (Figure 3, bottom panel). For these stimulator cells, a differential EV Array signal was particularly observed for HLA-DR. This marker was preferentially present, when analysing the EVs in the UC sample (blue), with an approximate 2.5 fold enrichment. A similar observation was demonstrated for CD63.

**Figure 4.**
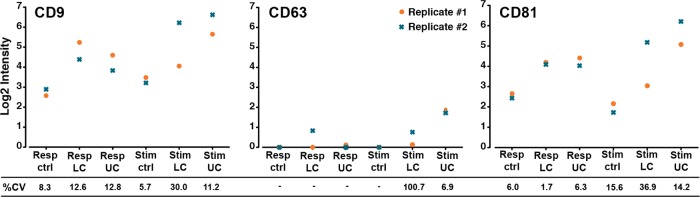
**Reproducibility of the cell-culturing set-up and analytical platform**. To demonstrate the reproducibility of the presented methodology, two independent experiments of the contact-independent co-culture were performed (using one biological replicate). The plots display the results from three selected EV markers; CD9, CD63 and CD81 (also shown in Figure 3] from the EV Array analysis of the resulting cell supernatants from the UC and the LC. The corresponding %CV values are noted below each sample, and were calculated based on the two technical replicates. The %CV values were not calculated for the samples with log2 signal intensities below the lower limit of detection (marked by a -).

As a final notion, the reproducibility of the presented cell-culturing set-up and analysis platform was evaluated. Accordingly, the contact-independent co-culture was repeated several weeks apart, using cells from the same individuals. In Figure 4, the results of three selected markers are presented for these two technical replicates. It can be seen that, for CD9 and CD81, the detected EV signals predominantly correlated from replicate to replicate, with 10 of the 12 obtained %CV values ranging from 1.7%-15.6% (Figure 4). The last two %CV values for CD9 and CD81 were 30% and 36.9%, and they were both calculated from the stimulator cells in the lower compartment. For CD63, four of the six %CV values could not be determined, as either one or both EV Array signals from the two technical replicates were below the lower limit of detection (LOD). The last two %CV values for CD63 were 6.9 and 100.7. For the latter sample, the detected log2 signals were very close to the lower LOD (log2 values: 0.13 and 0.75).

## 4. Discussion and Conclusion

In the research field of EVs, much effort is put into deciphering the biological functions of these vesicular entities. Consequently, useful experimental and analytical platforms are of great interest. In this technical report, we have investigated a commonly used cell-culturing system for the study of dynamic, contact-independent cell communication, focusing on the ability of the EVs involved to enter each compartment (depicted in Figure 1). In the applied set-up, a membrane with 0.4 μm pores separated the cells, but other pore sizes between 1–8 μm are also available. Initially, we wanted to investigate whether the transmembrane exchange of EVs were similar for both compartments. With the results presented in Figure 2, it is apparent that for this short study (<24h), the exchange of EVs was greater from the UC to the LC than in the opposite direction. The observed differential EV exchange is relevant because there are many studies investigating selected features of contact-independent cellular communication that have applied short incubation times [4, 8, 9, 12, 13]. Our study also suggests that this is important, since for short studies it may be advantageous to place the primary EV donor cells in the UC, while the primary recipient cells should be placed in the LC. These suggestions are based exclusively on the observations derived from Figure 2 and Table 1, since it was not determined which contributing factors could have affected this differential transmembrane EV exchange, such as diffusion, sedimentation or hydrostatic pressure. Moreover, the transmembrane EV exchange could have been investigated using a cell-containing set-up. Nevertheless, a cell-free approach was chosen since it incorporated the possibility of demonstrating any concentration dependency of the transmembrane EV exchange by making precise dilutions of the EV-rich supernatant. This option was not given when using cells, as it has not been confirmed that there is a linear correlation between the number of cells and the amount of EVs produced. One element that may alleviate any possible effects of the observed differential EV transport is agitation of the culturing set-up. However, this was not investigated in the current study, as agitation has not been employed in several other studies investigating contact-independent cell communication [4, 8, 9, 12, 13]. Consequently, the presented data relate to the practice of many relevant studies. As a technical note, it can be seen in Table 1 that the mean size of vesicles detected in the compartment, which initially only contained growth medium, was variable (79.3 nm versus 97.1 nm, respectively). When addressing the corresponding EV Array signals (4.3 versus 0.5, respectively), it has previously been demonstrated that vesicles that bind to the array are primarily <100 nm [11]. However, despite this reported size maximum, the mean size of the eluted EVs from the array in reality had a mean size <85 nm. Hence, for the present study, this correlates with the observation that the smaller sized vesicles yielded a higher amount of detected CD9, CD63 and CD81.

The differential transport of EVs across the insert membrane was observed in a simplified version of the cell-culturing system, which does not incorporate the dynamics of a cell-based set-up, where EVs are continuously produced and taken up by cells. However, the observed difference may be a contributing factor in the cell-based system as well. This might possibly be connected to the differences detected for the phenotype of the cell-derived EVs, which was affected by the relative placement of the two cell populations (Figure 3). Biological variations alone could, most likely, not account for this phenotypic difference, since the set-up was reproducible (Figure 4). Hence, this points to other determining factors, such as a differential transmembrane EV exchange. Moreover, the induced EV phenotypes in the co-cultures were not artefactual, as all investigated markers were detected above control levels. The cellular phenotype, which was investigated for a subset of the responder cells, was not affected to the same degree as the vesicular phenotype by the relative placement of the cell populations (data not shown). Any biological relevance of these observations has not yet been investigated in more detail. However, the EV phenotype may be more sensitive to the experimental design, with a possible importance of the functional consequences of the EV-based communication between the involved cell populations. Consequently, this stresses the point that it may not be trivial how the cell populations are placed relative to each other, both for short and long studies of contact-independent cell communication. Several studies do not account for their choice of the relative placement of the cells in the two compartments [4, 8, 9, 12, 13], which our study indicates as being relevant. As a final note, the recommendations, which are made using the presented data, are based on a co-culture of PBMCs. This cellular population consists predominantly of suspension cells with a smaller fraction of adherent cells. Therefore, applying other cell types with different characteristics could entail other affecting factors, such as an increased blocking of the membrane pores. Nevertheless, this yet again underlines the significance for investigating any influence that the relative placement of the cells has on the experimental outcome, regardless of the cell types used.

As mentioned previously, it is essential to have reliable assays to delineate the biology and functionality of EVs. Therefore, the reproducibility of the cell-based experiments was evaluated by repeating the co-cultures and the subsequent analysis after several weeks, using cells from the same individuals. As presented in Figure 4, the obtained %CV values for CD9 and CD81 ranged from 1.7 to 36.9. However, 10 of these 12 samples had a %CV below 16, whereas the two highest values were calculated for the same sample, namely the stimulator cells in the LC, pointing to an isolated trend for this sample. It has been established that the EV Array yields %CV values below 10 when working with plasma samples [10], and %CV values below 25 have been proposed as an acceptable limit for immunoassays [14]. However, this limit can be expanded if an experimental rationale is present [14], which may be the case when both technical and biological variations exist, as in this study. Nonetheless, one of the %CV values for CD63 differed to a somewhat greater extent, amounting to 100.7 (Figure 4). The log2 intensities from the relevant replicates, forming the basis of the %CV calculation, were 0.13 and 0.75. Hence, the standard deviation was very large compared to the mean of the two intensity values, with a consequent large impact on the calculated %CV. This is an inherent issue for small intensities close to the lower LOD, which is difficult to completely circumvent. A third technical replicate may serve to improve the %CV. However, several other results from our work point to the fact that CD63 is a poor marker for EVs in general since it occurs in relatively small amounts, as compared to CD9 and CD81 (unpublished data and [10, 11, 15–17]).

As part of the combined experimental set-up presented here, the determination of the EV phenotype plays a major role. Using the EV Array to phenotype the vesicles provides the opportunity to gain much information about the system that is being studied. In the context of vesicle-based cell communication, the EV phenotype may be used for several purposes. First, the use of an extensive EV phenotype may aid in fine-tuning the biological hypothesis that is being investigated. In this study, we targeted 11 protein markers. However, 60 analytes (in this case, antibodies) can currently be used simultaneously for each sample when phenotyping EVs with the EV Array [10]. Consequently, the EV phenotype may not only provide a large amount of information but it can also be used to optimize the experimental design in an iterative fashion. Furthermore, when applying the EV Array, this can be achieved without having to resort to extensive EV isolation procedures. Currently, the designed EV Array does not provide direct information about which cells produce the EVs and their absolute quantities. However, by linking the extensive EV phenotype to a number of additional experimental outcomes, such as the cellular phenotype, or the EV RNA and subproteome cargo, unravelling the biological functions of the EV-based communication becomes substantial and highly relevant.
